# Exploring a Sustainable Process for Polyphenol Extraction from Olive Leaves

**DOI:** 10.3390/foods13020265

**Published:** 2024-01-15

**Authors:** Nils Leander Huamán-Castilla, Karla Syndel Díaz Huamaní, Yolanda Cristina Palomino Villegas, Erik Edwin Allcca-Alca, Nilton Cesar León-Calvo, Elvis Jack Colque Ayma, Franz Zirena Vilca, María Salomé Mariotti-Celis

**Affiliations:** 1 Escuela de Ingeniería Agroindustrial, Universidad Nacional de Moquegua, Prolongación Calle Ancash s/n, Moquegua 18001, Peru; karla03011988@gmail.com (K.S.D.H.); yolacristi@hotmail.com (Y.C.P.V.); eallccaa@unam.edu.pe (E.E.A.-A.); nleonc@unam.edu.pe (N.C.L.-C.); 2 Laboratorio de Tecnologías Sustentables para la Extracción de Compuestos de Alto Valor, Instituto de Investigación para el Desarrollo del Perú (IINDEP), Universidad Nacional de Moquegua, Prolongación Calle Ancash s/n, Moquegua 18001, Peru; 3 Laboratorio de Contaminantes Orgánicos y Ambiente, Instituto de Investigación para el Desarrollo del Perú (IINDEP), Universidad Nacional de Moquegua, Moquegua 18001, Peru; colqueaymajack@gmail.com (E.J.C.A.); fzirenav@unam.edu.pe (F.Z.V.); 4 Escuela de Nutrición y Dietética, Facultad de Medicina, Universidad Finis Terrae, Santiago 7501015, Chile

**Keywords:** olive leaves, polyphenols, antioxidant capacity, pressurized liquid extraction, green solvent

## Abstract

Olive leaves are residues from pruning and harvesting and are considered an environmental management problems. Interestingly, these residues contain high polyphenol concentrations, which can be used to treat chronic diseases. However, these compounds are a technological challenge due to their thermolability and reactivity during extraction. Thus, this study assessed the use of pressurized liquid extraction (PLE) with green solvents like water-ethanol and water-glycerol mixtures (0–15%) at 50 °C and 70 °C to yield polyphenol-rich antioxidant extracts with reduced glucose and fructose content. The use of 30% ethanol at 70°C presented the highest polyphenol content (15.29 mg gallic acid equivalent/g dry weight) and antioxidant capacity, which was expressed as IC_50_ (half maximal inhibitory concentration): 5.49 mg/mL and oxygen radical absorbance capacity (ORAC): 1259 μmol Trolox equivalent/g dry weight, as well as lower sugar content (glucose: 3.75 mg/g dry weight, fructose: 5.68 mg/g dry weight) compared to water–glycerol mixtures. Interestingly, ethanol exhibits a higher degree of effectiveness in recovering flavanols, stilbenes and secoiridoids, while glycerol improves the extraction of phenolic acids and flavonols. Therefore, to enhance the efficiency of polyphenol recovery during the PLE process, it is necessary to consider its solvent composition and chemical structure.

## 1. Introduction

The world’s olive industry cultivates 10.8 million hectares of this fruit, yielding a total production of 21.6 million tons [1]. In particular, Peru has approximately 21,000 hectares dedicated to cultivating this fruit, with Arequipa, Tacna, and Moquegua representing 73%, 20%, and 5% of the cultivated area of this fruit, respectively [2]. Olive is a product abundant in unsaturated fatty acids like oleic, linoleic, and linolenic, whose direct consumption offers various health benefits to people [3,4]. However, obtaining olives involves several preceding stages, including flowering, fruit development, pit hardening, fruit ripening, and the accumulation of oil [5]. These stages generate significant quantities of olive tree leaves, an agro-industrial waste with no economic value. They are considered an environmental management problem due to the formation of greenhouse gases (methane) [6]. However, this waste is a natural source of polyphenols, attracting the attention of both the pharmaceutical and food industries due to its bioactive properties [7].

Under stressful conditions, plants synthesize polyphenols as a defense mechanism through the shikimic acid pathway to mitigate free radicals within their biological system [8,9]. Polyphenol’s chemical structure presents hydroxyl groups and phenolic rings [10]. In particular, hydroxyl groups can donate electrons to neutralize free radicals; this electron donation process defines the antioxidant capacity of polyphenols [11,12]. This property to scavenge free radicals significantly protects cells and tissues from oxidative stress-related damage [13]. Olive leaves contain important concentrations of total polyphenols (35–100 mg equivalent gallic acid (GAE)/g), which present different families of specific polyphenols such as secoiridoids, flavones, flavanols, flavonols and phenolic acids [14,15,16]. The bioactive properties of these specific compounds are related to treating and preventing diseases associated with oxidative stress. For example, Oleuropein is a secoiridoid that has the capability to induce growth arrest or death in cancer cells due to its ability to inhibit the activity of enzymes within the mitochondria [17]. Catechin (flavanol) can impact bacterial cell membranes; this property alters these microorganisms’ functional aspects and growth [18]. Quercetin (flavonol) exhibits the ability to modulate inflammation by inhibiting inflammatory enzymes such as cyclooxygenase and lipooxygenase [19]. Gallic acid (phenolic acid) has exhibited promising potential in treating obesity by regulating anti-inflammatory mediators (adipocytes and macrophages) [20]. Thus, finding a sustainable method to recover the polyphenols in olive leaves is still a challenge that requires resolution.

Several research studies have explored different conventional extraction methods at atmospheric conditions to recover polyphenols [21]. These methods use organic solvents like acetone, methanol, and hexane, demonstrating their ability to obtain extracts rich in polyphenols from vegetable matrices [22,23]. For example, acetone has a greater ability to interact with polyphenols due to its solvent’s ability to accept the solute’s protons during the extraction process [24,25,26]. Thus, water–acetone mixtures (60%) have been proposed as the most efficient solvent for extracting polyphenols under atmospheric conditions [27,28,29]. However, these methods are not food grade and involve extended processing times exceeding 4 h. They promote the hydrolysis and oxidation of these compounds, thereby restricting their industrial applicability [30]. Thus, the development of alternative technologies to produce food ingredients using food-grade solvents is desirable.

Pressurized liquid extraction (PLE) is an alternative to conventional extraction methods. Under subcritical conditions, this technique involves the use of pure water or its combinations with solvents like ethanol, glycerol, or isopropanol to enhance the extractability of polyphenols within short processing times (<20 min) and using minimal solvent volumes [31,32,33,34]. PLE is a method conducted under subcritical conditions, wherein food-grade solvents operate at temperatures and pressures below their critical points; this specific environment enables solvents to retain their liquid form and reduces their dielectric constant, density, viscosity and surface tension, increasing the transfer rates of compounds from the matrix to the solvent [35,36]. Simultaneously, an increase in temperature increases the kinetic energy of the solvent molecules breaking the matrix [34]. Thus, this process enhances the extractability and solubility of bioactive compounds.

In PLE, different factors impact the efficacy of this method to recover polyphenols such as plant matrix, the polarity of the solvent, temperature, the chemical structure of the polyphenols, and the presence of interfering compounds (sugars, fibers, and toxic substances) [37,38,39]. For example, when the temperature of the water is increased from 20 to 275 °C, its polarizability (π*) decreases to 0.69, which is similar to the polarizability of methanol (π*: 0.59) and ethanol (π*: 0.53) at 30 °C [37,40]. Thus, during PLE, water exhibits comparable effectiveness to methanol and ethanol in extracting polyphenols. On the other hand, using 15% ethanol at 90 °C has been more effective in recovering polyphenols than using pure water at 130 °C [41]. In addition, adjusting the solvent composition using 15%, 32.5%, and 50% ethanol during the ELP at 150 °C has enabled a selective process to recover flavonols, flavanols, and phenolic acids, respectively [31]. However, elevated process temperatures (T° > 150 °C) during PLE could result in the degradation and hydrolysis of polyphenols, as well as the creation of potentially carcinogenic Maillard compounds, including hydroxymethylfurfural (HMF). Nonetheless, the incorporation of alternative co-solvents like ethanol (15%) at lower temperatures (<90 °C) not only holds promise in increasing polyphenol recovery yields but will also allow a reduction of the presence of reducing sugars, which act as precursors to HMF formation [41].

To date, some studies have incorporated ethanol and glycerol in the extraction process to enhance the overall recovery of polyphenols. Nevertheless, these investigations have primarily focused on optimizing the extraction of polyphenols alone, overlooking the recovery of undesired compounds like sugars. Thus, this work carries out an exhaustive analysis of the extraction process parameters to understand the impact of co-solvents on the recovery and selectivity of antioxidant compounds and the reduction of sugars.

## 2. Materials and Methods

### 2.1. Samples

OLIVERS company S.A.C, situated in Peru’s Moquegua Region, supplied 10 kg of olive leaves. These leaves were obtained from the pruning process. They were carefully enveloped in white tissue paper for protection and placed in corrugated cardboard boxes to shield them from light exposure and physical harm. Subsequently, a grinder (MS6CA4120 ErgoMixx 800W, Bosch, München, Germany) was used to reduce the samples to a particle size of 2 mm.

### 2.2. Chemical Reagents

Sigma Aldrich Chemical Co. (St. Louis, MO, USA) supplied reducing sugars (fructose and glucose), Folin-Ciocalteu reagents, DPPH radical (2,2-Diphenyl-1-picrylhydrazyl), AAPH (2,2′-azobis (2-methyl-propanimidamide) dihydrochloride), fluorescein, and Trolox, as well as target polyphenols like caffeic (≥97%), quercetin (≥95%), epicatechin (≥98%), resveratrol (≥98%), catechin (≥97%), kaempferol (≥97%), oleuropein (≥98%), gallic (≥95%), and vanillic (≥95%), while methanol (≥99%) and ethanol (≥99%) were provided by J.T. Baker Chemical Co. (Temixco, Mexico).

### 2.3. Extraction Method to Recover Polyphenols

The PLE method was employed to extract polyphenols from olive leaves. This was achieved by utilizing different solvent mixtures, including water–glycerol (from 0% to 30% *w*/*w*) and water–ethanol (from 0% to 30% *w*/*w*) combined with varying temperatures of extraction (50 °C and 70 °C). The solvent levels and process parameters were selected by prior research [31,32]. Then, an extraction cell was used to load ~10 g of dried olive leaves and 40 g of neutral quartz sand, which were mixed previously. For extraction, a pressurized liquid system (ASE 150, Dionex, Thermo Fisher, San Jose, CA, USA) was used combined with the following parameters: 10 MPa pressure, a single extraction cycle, 150% wash volume, 250 s of nitrogen purge time, and a static extraction duration of 5 min. The obtained extracts were filtered and stored at −20 °C.

### 2.4. Total Polyphenol Content (TPC)

The obtained extracts were analyzed using the methodology established by Singleton et al. [42] with some modifications. A total of 0.25 mL of extract, 3.75 mL of pure water, 0.25 mL of Folin-Ciocalteu reagent (1N), and 0.5 mL of sodium carbonate (10% *w*/*v*) were mixed. Afterwards, a spectrophotometer Genesys 150 (Thermo Fisher, San Jose, CA, USA) was used to measure the total polyphenol content at 765 nm after the mixture was protected from light for 1 h at 20 °C. The results were expressed as milligrams of Gallic Acid Equivalent (GAE) per gram of dry weight. Simultaneously, a calibration curve was obtained measuring different concentrations of Gallic Acid standards at the same wavelength.

### 2.5. Antioxidant Capacity by 2,2-Diphenyl-1-picrylhydrazyl (DPPH) Analysis

The Brand-Williams method [43] was used to evaluate the antioxidant capacity of extracts. A mixture of different dilutions of extract (0.1 mL) combined with 3.9 mL of DPPH solution (0.1 mM) was protected from light for 30 min at room temperature. This allows for the reaction between the antioxidant compounds in the extract and the DPPH radical. After 30 min, a spectrometer (Genesys 150 UV Spectrometer, Thermo Fisher, San Jose, CA, USA) measured the reaction at 517 nm. The results were expressed as IC_50_, the concentration of extract rich in polyphenols necessary to scavenge 50% of the DPPH solution. This value was obtained from linear regression (R^2^: 0.9997), which suggests that the relationship between the concentration of the antioxidant compound and the inhibition of DPPH radical activity follows a linear trend.

### 2.6. ORAC Analysis to Determine Antioxidant Capacity

The methodology was described by Chirinos et al. [44]. This follows the Oxygen Radical Absorbance Capacity (ORAC) assay, which measures the antioxidant capacity of substances against peroxyl radicals. PBS buffer (pH 7.4) prepared 8 nM fluorescein and 153 nM AAPH solutions. Then, different concentrations of the extract or Trolox solution were mixed with fluorescein and AAPH solutions in a microplate. The samples were incubated at 37 °C for 10 min. After incubation, the microplate was placed in the reader (Synergy/HTX, Biotek Instruments Inc., Winooski, VT, USA), which was configured with the appropriate excitation and emission wavelengths (485 nm excitation, 520 nm emission). The ORAC results were obtained by plotting the fluorescence values against time and integrating the area under the curve. Simultaneously, Trolox calibration curves with known concentrations convert the area under the curve to Trolox equivalents. These results were expressed as µmol Trolox equivalents per gram of dry weight.

### 2.7. Analysis of Reducing Sugars (Glucose and Fructose)

The extracts were analyzed to quantify glucose and fructose content based on the methodology outlined by Mariotti et al. [41]. The extracts were prepared and diluted with methanol and standard solutions of known concentrations of glucose and fructose to create a calibration curve. Afterwards, an HPLC system (Ultimate 3000, Dionex Thermo Scientific, Sunnyvale, CA, USA) with a normal phase column (Li ChroCART 250-4 Purospher STAR, 5 μm) was configured under isocratic conditions like mobile phase (acetonitrile solution at 70%) and flow rate of 1 mL/min. Then, the samples prepared were injected (20 µL) into the HPLC system to generate a calibration curve relating the concentration of reducing sugars to their chromatographic peak areas. The results were expressed as milligrams of specific reducing sugar per gram of dry weight.

### 2.8. Quantification of Target Polyphenols

The extracts were purified using a solid-phase extraction method (HyperSep™ C18 Cartridges, Thermo Scientific, Waltham, MA, USA). Then, an HPLC system (Agilent 1290 II, Santa Clara, CA, USA) was used to quantify polyphenols with a UV-Vis or photodiode array detector reverse and phase Poroshell 120 Ec-C18 column (2.1 × 150 mm × 1.9 µm).

The HPLC system was configured at 30 °C and 0.3 mL/min. At the same time, gradient elution consisted of two mobile phases, A (acetonitrile and formic acid 0.1%) and B (water and formic acid 0.1%), and was configured with 95% A and 5% B for 15 min, followed by 60% A and 40% B for 18 min, and finally, 95% A and 5% B for 20 min.

Samples were injected (2 µL) into the HPLC system to separate polyphenolic peaks and determine their quantities. Before analysis, standard solutions containing specific polyphenols were injected to establish calibration curves (Table 1). Calculations of polyphenol concentrations relied on the peak areas obtained from the chromatograms and the established calibration curves. The results were expressed as micrograms of specific polyphenols per dry gram of dry weight.

### 2.9. Statistical Analysis

A full factorial experimental design was executed to assess the effect of studied factors, such as solvent composition and extraction temperature, on response variables (polyphenol content, antioxidant capacity, and sugar-reducing properties). This method enabled the exploration of all possible combinations of factor levels to comprehensively understand their individual and combined effects on the response variables. Subsequently, Analysis of Variance (ANOVA) was employed to compare means and ascertain statistically significant differences among the observed groups (*p*-value < 0.05). Following the ANOVA, Tukey’s Honestly Significant Difference (HSD) test was conducted for multiple pairwise comparisons between group means and identified significant differences. Statgraphics Plus for Windows 4.0 (Statpoint Technologies, Inc., Warrenton, VA, USA) was used to analyze the data.

## 3. Results and Discussion

### 3.1. Effect of Alternative Solvents during PLE

Pressurized liquid extraction presents distinct advantages, including heightened efficiency and improved yields in contrast to conventional extraction methods conducted under atmospheric conditions. However, it is important to note that extraction parameters, such as solvent composition and temperature, can vary based on the specific characteristics of the plant matrix, which can affect the concentration of antioxidant compounds and reduce sugar content.

#### 3.1.1. Total Polyphenol Content

**Figure 1:**
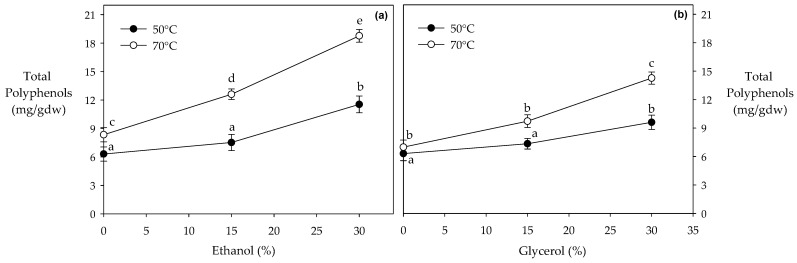
Recovery of polyphenol at different temperatures and solvent composition. Different letters indicate statistically significant differences (*p* < 0.05). (**a**,**b**) represent the polyphenol content when ethanol and glycerol were used as solvents in the PLE, respectively.

According to our results, as temperature and solvent concentration increase, there is a significant increase in the recovery of polyphenols (Figure 1). Interestingly, the use of ethanol as an alternative solvent at high temperatures was significantly more efficient in recovering important concentrations of phenolic compounds compared to glycerol and pure water (Figure 1a,b). For example, 30% ethanol at 70 °C improved the recovery of polyphenols by 31% and 136% compared to 30% glycerol and pure water under the same conditions, respectively (Figure 1a,b).

Some studies have reported the efficacy of utilizing ethanol under subcritical conditions. For example, Rosa et al. [45] observed that using 80% ethanol at 60 °C allowed the recovery of 48% more polyphenol content compared to using pure water under the same conditions. Mariotti-Celis et al. [41] reported that using 15% ethanol at 60 °C improved the recovery of polyphenols by 37% compared to pure water at 60 °C. Xynos et al. [46] observed that using 100% ethanol at 40 °C increased the recovery of 48% more polyphenol content compared to using water under the same conditions. Although the water molecule favors polar interactions with the hydroxyl groups of polyphenols, ethanol possesses both polar and non-polar groups within its chemical structure, favoring intermolecular interactions not only with hydroxyl groups but also with the aromatic groups of polyphenols [24,31].

Although ethanol and glycerol are solvents commonly used in extracting polyphenols, ethanol can be considered a better solvent for the recovery of these compounds due to the dual polarity present in ethanol’s chemical structure (aromatic and hydroxyl groups), which affords its greater efficiency in the recovery of these bioactive compounds compared to glycerol, which presents three hydroxyl groups [31,32]. In addition, ethanol has a higher polarity than glycerol, making it more effective in dissolving a wide range of polyphenols [25,47]. Finally, ethanol is generally considered safe (GRAS) for food and pharmaceutical applications. It is widely accepted and regulated as a solvent for extracting bioactive compounds, including polyphenols, making it a preferred choice in industries adhering to strict regulatory standards. However, it is important to note that the choice of solvent depends on various factors, such as the specific polyphenols being targeted, the characteristics of the plant material, and the particular applications.

#### 3.1.2. Antioxidant Capacity

Oxygen Radical Absorbance Capacity (ORAC) and 2,2-diphenyl-1-picrylhydrazyl (DPPH) assays are used to measure the antioxidant capacity of compounds in the obtained extracts. However, the ORAC analysis is often regarded as more related to the physiological system due to its ability to evaluate polyphenols’ effectiveness in neutralizing peroxyl radicals, which are similar to the free radicals generated within our bodies [48,49]. At the same time, the DPPH method assesses the capacity of polyphenols to reduce the DPPH radical, a distinct free radical differing from other biologically produced reactive species. Thus, IC_50_ is expressed as mg of extract to inhibit 50% of the DPPH radical solution (mL).

**Figure 2:**
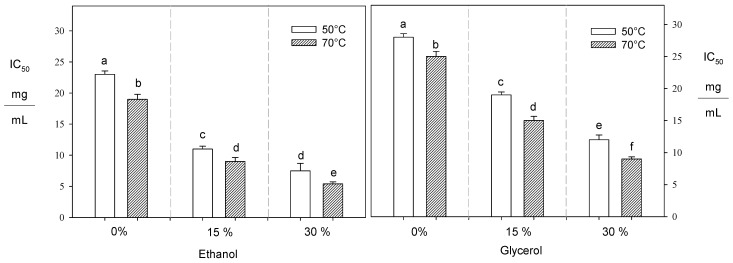
Effect of temperature and solvent composition on IC_50_ value. Different letters indicate statistically significant differences (*p* < 0.05).

For the DPPH method, when the temperature increased from 50 to 70 °C with ethanol (30%), glycerol (30%), and pure water, the amount of extract necessary to inhibit the DPPH radical was reduced by 29%, 23%, and 16%, respectively (Figure 2). Xynos et al. [46] observed that the temperature was increased from 40 to 62 °C using water–ethanol mixtures (50%) to reduce the necessary extract volume to inhibit the DPPH radical from olive leaves by 14%. Young et al. [50] reported that an increment in ethanol concentration from 0% to 30% reduced by 16% the required extract amount to reduce the DPPH radical from Dendropanax morbifera leaves. Under subcritical conditions, an increase in temperature enhances the solvent’s kinetic energy, facilitating the plant matrix’s breakdown. Consequently, more polyphenols are released to interact with the DPPH radical [41]. In this sense, using alternative solvents like ethanol and glycerol improves the intermolecular interactions between the functional groups of the solvent and the polyphenol [31,32,51].

**Figure 3:**
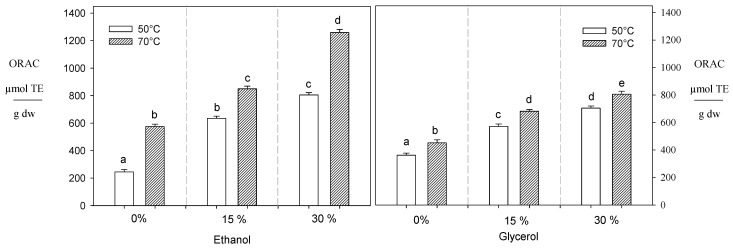
Effect of temperature and solvent composition on ORAC value. Different letters indicate statistically significant differences (*p* < 0.05).

On the other hand, the extracts obtained using 30% ethanol, 30% glycerol, and pure water at 70 °C exhibited the highest ORAC values, measuring 1259, 731, and 576 µMTE/g dw, respectively (Figure 3). In contrast to the DPPH results, the ORAC values directly correlated with the polyphenol content. Thus, the greater the ORAC antioxidant capacity, the higher the concentration of polyphenols present in the extracts obtained.

#### 3.1.3. Sugar Reducing

**Figure 4:**
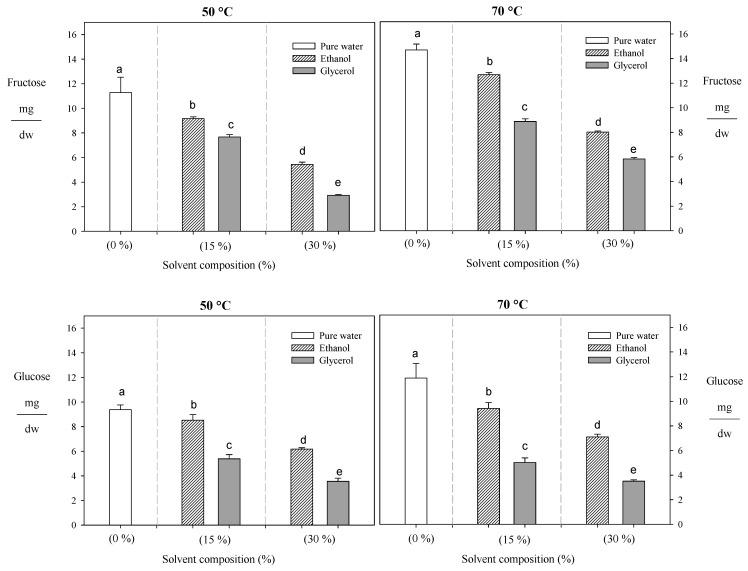
Effect of temperature and solvent composition during PLE on the recovery of reducing sugars. Different letters indicate statistically significant differences (*p* < 0.05).

Although an increase in temperature enhances sugar’s recovery, using alternative solvents reduces these compounds’ extractability (Figure 4). For example, when the ethanol increased from 0% to 30% at 70 °C, it led to a decrease in the levels of fructose and glucose by 34% and 40%, respectively, while when glycerol (from 0% to 30%) was used the fructose and glucose content decreased by 77% and 70% under the same conditions, respectively (Figure 4). Similar behavior was reported by Huaman et al. [34], who observed that adding ethanol from 15% to 50% reduces the extraction of reducing sugars (glucose and fructose) by 60%. This phenomenon can be attributed to using intermediate polarity solvents like ethanol during the extraction process, which diminishes the ability of these polar compounds (reducing sugars) to form hydrogen bonds with water molecules [52]. Consequently, a lower concentration of these compounds is recovered in the extracts. On the other hand, glycerol exhibited greater efficacy in reducing the presence of reducing sugars compared to ethanol. This could probably be attributed to glycerol’s structural composition, which incorporates three hydroxyl groups, likely providing an enhanced capacity to establish hydrogen bonds with water molecules. This minimizes interactions between reduced sugars and water.

### 3.2. Impact of Solvent Composition on the Recovery of Specific Polyphenols

**Figure 5:**
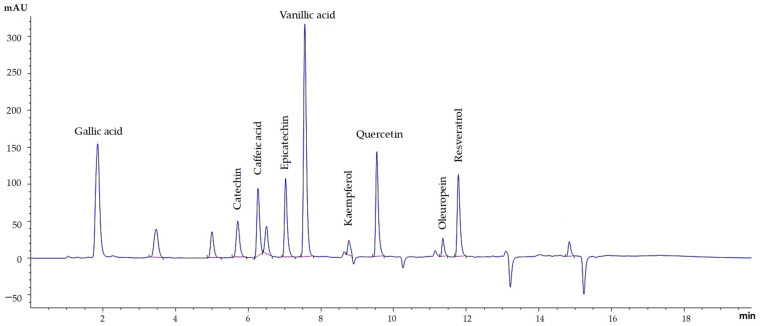
UHPLC chromatogram of nine standards of specific polyphenols.

Liquid chromatography was employed to analyze various specific polyphenols to comprehend how the solvent’s polarity influences the recovery of antioxidant compounds (Figure 5). According to our results, At the highest solvent concentration (30%), the highest temperature (70 °C) recovered more specific polyphenol content than the lowest temperature (50 °C). For phenolic acids, using 30% glycerol allowed the recovery of 42% more phenolic acids compared to using ethanol under the same conditions (Table 2). A similar behavior was reported by Huaman et al. [32], who observed that 50% glycerol at 150 °C increased the recovery of phenolic acids by 53% compared to the use of 50% ethanol under the same conditions. The best condition (glycerol 30%—70 °C) allowed the quantification of three phenolic acids such as gallic, caffeic and vanillic, where gallic acid was the predominant polyphenol with (~92 µg/g dw).

Similarly, to phenolic acids, the recovery of flavanols at high temperatures (70 °C) was improved when 30% glycerol was used as solvent extraction (396 µg/g dw) compared to the use of 30% ethanol (281 µg/g dw) (Table 2). Glycerol is a polar compound due to three hydroxyl groups, which can interact with carbonyl and hydroxyl groups in the flavanol’s chemical structure [32,33]. Under these conditions, quercetin and kaempferol were quantified with 194 µg/g dw and 202 µg/g dw, respectively.

Although phenolic acids and flavonols had a higher extraction using glycerol due to their polarity and compatibility, the recovery of flavonols and stilbenes was more efficient when ethanol was utilized as the extraction solvent (Table 2). For example, the 30% ethanol solution was 29% and 43% more effective in extracting flavanols and stilbenes than the 30% glycerol solution (Table 1). Probably, ethanol’s bipolar nature due to the presence of a polar (hydroxyl group) and non-polar (methyl) fraction increases the intermolecular interactions with the functional groups of flavanols and stilbenes (hydroxyl and phenolic group) [24,31]. The best condition (ethanol 30%—70 °C) allowed the recovery of important concentrations of catechin (176 µg/g dw) and resveratrol (94 µg/g dw).

On the other hand, ethanol allowed a higher recovery of secoiridoids compared to glycerol. For example, when 30% ethanol was used at 70 °C, the recovery of oleuropein improved by 30% compared to 30% glycerol under the same conditions (Table 1). Under these conditions (ethanol 30%—70 °C), the recovery of this compound reached 1312 µg/g dw. Oleuropein stands out as the predominant polyphenol found in olive leaves; consequently, there is considerable interest within the food industry in identifying and establishing the most effective process conditions for its extraction.

## 4. Conclusions

Regardless of the solvent type, elevated temperatures (70 °C) during ELP facilitated the recovery of both polyphenols and reducing sugars from olive leaves. However, when alternative solvents like glycerol (30%) and ethanol (30%) were used during ELP at 70 °C, the extraction of antioxidant compounds improved by 65 and 40% compared to pure water. In comparison, the recovery of sugars (glucose and fructose) was reduced by between 34 and 77%. Ethanol has two fractions (polar and non-polar) in its chemical structure, which can interact with the hydroxyl and aromatic groups of the polyphenols. On the other hand, the liquid chromatography analysis of the obtained extracts showed that ethanol exhibits a higher degree of effectiveness in recovering flavanols, stilbenes and secoiridoids. At the same time, glycerol improves the extraction of phenolic acids and flavonols. Therefore, temperature and the solvent’s polarity are key factors in achieving an efficient extraction process capable of obtaining antioxidant-rich extracts with reduced sugar concentrations. These findings provide valuable insights that can be applied to scale up industrial processes to recover bioactive compounds from diverse plant matrices.

## Figures and Tables

**Table 1:** Analytical parameters for the quantification of specific polyphenols.

Specific Polyphenol	Wavelength (nm)	Regression Equation	R^2^
Oleuropein	254	Y = 59.2803X − 0.7963	0.9999
Gallic	270	Y = 6.615X + 1.7801	0.9992
Quercitin	270	Y = 24.4618X + 2.282	0.9997
Caffeic acid	270	Y = 149.1198X + 0.9753	0.9999
Catechin	280	Y = 25.2511X − 0.5309	0.9999
Epicatechin	280	Y = 43.3950X − 2.1554	0.9998
Vanillic acid	280	Y = 141.9918X − 5.4568	0.9998
Resveratrol	324	Y = 78.8100X − 3.3578	0.9997
Kaempferol	373	Y = 38.0226X − 1.5363	0.9997

**Table 2:** Polyphenols profile present in the obtained extracts.

	50 °C					70 °C				
	Pure Water	15%		30%		Pure Water	15%		30%	
Phenolic Acids		Ethanol	Glycerol	Ethanol	Glycerol		Ethanol	Glycerol	Ethanol	Glycerol
Caffeic	11.1a ±2.2	22.5b ±3.2	19.2b ±2.2	28.5c ±5.1	23.4b ±1.7	16.1b ±1.7	31.3c ±2.7	28.3c ±1.6	43.3d ±3.9	33.4c ±1.9
Vanillic	15.1a ±4.3	21.4a ±3.3	23.3a ±4.3	46.4d ±3.9	36.9c ±4.5	21.1a ±4.3	28.7b ±2.1	25.2a ±2.5	56.2e ±2.4	47.6d ±3.8
**Stilbenes**										
Resveratrol	33.5a ±3.3	42.9a,b ±2.1	35.9a ±4.1	66.7d ±3.9	55.1c ±4.7	38.4a ±6.3	54.9 ±5.2	46.9b ±3.3	94.4e ±5.9	53.7c ±4.8
**Flavanols**										
Catechin	79.0a ±6.6	110.0c ±8.1	98.0b ±7.6	148.6d ±9.9	122.6c ±11.6	120.3c ±10.1	158.6e ±8.3	131.8d ±9.8	176.9f ±11.2	156.5e ±7.6
Epicatechin	69.7a ±5.8	88.4a ±6.7	76.7a ±8.3	112.7b ±8.4	91.8a ±5.9	95.3b ±7.1	129.2c ±10.2	106.8b ±9.1	152.8d ±12.4	112.8b ±9.8
**Flavonols**										
Quercetin	59.6a ±3.9	78.2b ±6.8	118.8d ±5.8	61.2a ±7.8	147.9e ±10.8	91.4c ±6.2	131.7e ±9.8	155.5e ±11.8	118.2d ±12.7	194.4f ±10.8
Kaempferol	30.0a ±4.6	72.9c ±4.7	121.5d ±9.5	59.6b ±6.7	158.3e ±11.2	79.9c ±7.8	125.2d ±10.2	162.4e ±9.8	102.9d ±8.8	202.5f ±11.6
**Secoiridoids**										
Oleuropein	417.5a ±24.6	522.6c ±33.6	457.8b ±14.7	620.5d ±12.4	595.8c ±18.3	563.6c ±44.7	960.3e ±34.4	810.2d ±24.3	1312.5f ±28.6	949.9g ±44.6

The table shows the mean and standard deviation (n: 3). Different letters indicate statistically significant differences (*p* < 0.05).

## Data Availability

Data is contained within the article.
